# Speech Processing to Improve the Perception of Speech in Background Noise for Children With Auditory Processing Disorder and Typically Developing Peers

**DOI:** 10.1177/2331216518756533

**Published:** 2018-02-14

**Authors:** Sheila Flanagan, Tudor-Cătălin Zorilă, Yannis Stylianou, Brian C. J. Moore

**Affiliations:** 1 Department of Experimental Psychology, University of Cambridge, UK; 2 Toshiba Research Europe Ltd., Cambridge Research Laboratory, UK; 3 Department of Computer Science, University of Crete, Heraklion, Greece

**Keywords:** speech intelligibility, speech enhancement, auditory processing disorder (APD), children, speech in noise

## Abstract

Auditory processing disorder (APD) may be diagnosed when a child has listening difficulties but has *normal* audiometric thresholds. For adults with normal hearing and with mild-to-moderate hearing impairment, an algorithm called spectral shaping with dynamic range compression (SSDRC) has been shown to increase the intelligibility of speech when background noise is added after the processing. Here, we assessed the effect of such processing using 8 children with APD and 10 age-matched control children. The loudness of the processed and unprocessed sentences was matched using a loudness model. The task was to repeat back sentences produced by a female speaker when presented with either speech-shaped noise (SSN) or a male competing speaker (CS) at two signal-to-background ratios (SBRs). Speech identification was significantly better with SSDRC processing than without, for both groups. The benefit of SSDRC processing was greater for the SSN than for the CS background. For the SSN, scores were similar for the two groups at both SBRs. For the CS, the APD group performed significantly more poorly than the control group. The overall improvement produced by SSDRC processing could be useful for enhancing communication in a classroom where the teacher’s voice is broadcast using a wireless system.

## Introduction

Some children have difficulty in understanding speech in the presence of background sounds despite having a pure-tone audiogram within the normal range, which is usually taken as audiometric thresholds better than 20 dB HL over the range 0.5 to 4 kHz or 0.25 to 8 kHz. If such a child scores poorly on a test of auditory processing such as the SCAN-3:C (Keith, 2000) or the dichotic digits test (Musiek, 1983), then the child may be suspected of having an auditory processing disorder (APD). APD is a heterogeneous condition that may involve temporal, spectral, and binaural aspects of hearing (Moore, 2006). It is unclear whether the listening difficulties that are described for children with APD are because of sensory or cognitive impairments or a combination of the two. Also, some researchers have questioned whether APD is a distinct disorder (Dawes, Bishop, Sirimanna, & Bamiou, 2008; Moore, Rosen, Bamiou, Campbell, & Sirimanna, 2013). For example, Dawes et al. (2008) compared children diagnosed with APD and a group who had not been diagnosed with APD and found that the two groups were hard to distinguish on the basis of symptoms or aetiology. Dawes, Sirimanna, Burton, Vanniasegaram, Tweedy, and Bishop (2009) compared children diagnosed with APD and children diagnosed with dyslexia and found similar proportions with poor auditory performance in the two groups.

Despite the lack of consensus as to the nature of APD and whether APD is a distinct disorder, it is a fact that many children experience listening difficulties in everyday life. In particular, many children have difficulty in understanding the teacher in a classroom, especially when the classroom is noisy (Nelson & Soli, 2000). Children with hearing loss are often equipped with hearing aids that include a wireless receiver. The teacher wears a microphone that is close to their mouth and the signal picked up by the microphone is transmitted to the receiver on the child’s hearing aid(s), hence delivering a relatively *clean* signal that is almost free of reverberation (Mecklenburger & Groth, 2016). Similar systems have been used for children diagnosed with APD, although in such cases, amplification may not be required (Johnston, John, Kreisman, Hall, & Crandell, 2009). However, hearing-impaired children and children with APD may still experience difficulties when using such systems if there is background noise in the classroom, as is often the case (Nelson & Soli, 2000).

Speech intelligibility in classroom situations could potentially be improved by processing of the signal picked up by the teacher’s microphone prior to transmission of the signal to the child’s hearing aid(s). Several research groups have developed algorithms for processing speech so as to enhance its intelligibility when background noise and reverberation are added after the processing has been applied (Cooke, Mayo, & Valentini-Botinhao, 2013; Yoo et al., 2007; Zorila, Kandia, & Stylianou, 2012). It would be trivial to improve the intelligibility of speech simply by increasing its level, thereby improving the signal-to-background ratio (SBR). The improvement that could be produced in this way would be limited by loudness tolerance. Therefore, algorithms of this type have typically been evaluated under the constraint that the root-mean-square level of the speech should be the same before and after processing (Cooke et al., 2013; Zorila et al., 2012). More recently, some such algorithms have been evaluated under the constraint that the loudness of the speech should be the same before and after processing (Zorila, Flanagan, Moore, & Stylianou, 2016; Zorila, Stylianou, Flanagan, & Moore, 2017). The loudness equalization has been achieved using a loudness model (Glasberg & Moore, 2002; Zorila, Stylianou, Flanagan, & Moore, 2016).

Here, we evaluated the potential effectiveness of one such algorithm—spectral shaping with dynamic range compression (SSDRC; Zorila et al., 2012)—for a group of children with APD and for a control group of children without reported listening difficulties, using the equal-loudness constraint. The SSDRC algorithm has been shown to be effective for adults with normal hearing (Zorila, Flanagan, et al., 2016) and for adults with mild-to-moderate hearing loss (Zorila et al., 2017), but to our knowledge it has not previously been evaluated for normally developing children or children diagnosed with APD.

## Method

### Ethical Approval

The study was approved by the Psychology Research Ethics Committee of the University of Cambridge and was conducted in accordance with the Declaration of Helsinki. Written, informed consent was obtained from all participants and their parents/guardians.

### Participants

A total of 18 children took part in the study. They were paid for their time and travel expenses. All of the children were in mainstream education, in School Year Groups 5 to 10, with English as their native language. Eight of the participants (4 boys) had an existing diagnosis of developmental APD from an audiologist. The children were diagnosed based on parental complaint of a hearing difficulty, an audiogram within the normal range for at least one ear, and scores below the normal range for at least one of the four main subtests of the SCAN-3:C (Keith, 2000) screening test for APD. The subtests are auditory figure ground, competing words, filtered words, competing sentences, and time-compressed speech; see the Appendix for details. SCAN-3:C scores for the APD group are given in Table A1. Note that the scores for three (1, 2, and 4) of the children in the APD group would be classified as borderline. One child (apd8) had been described by a specialist tutor as having “a moderate probability of dyslexia.” The other children in the APD group had not been diagnosed with any problem other than APD. The average age of the APD group at the time of the study was 12 years 1 month (range: 9 years 11 months–15 years 8 months). The control group was made up of 10 age-matched typically developing children (7 boys) with no reported listening, developmental, language, cognitive, or behavioral problems. Their average age was 12 years 5 months (range: 9 years 9 months–15 year 9 months).

**Figure 1.**
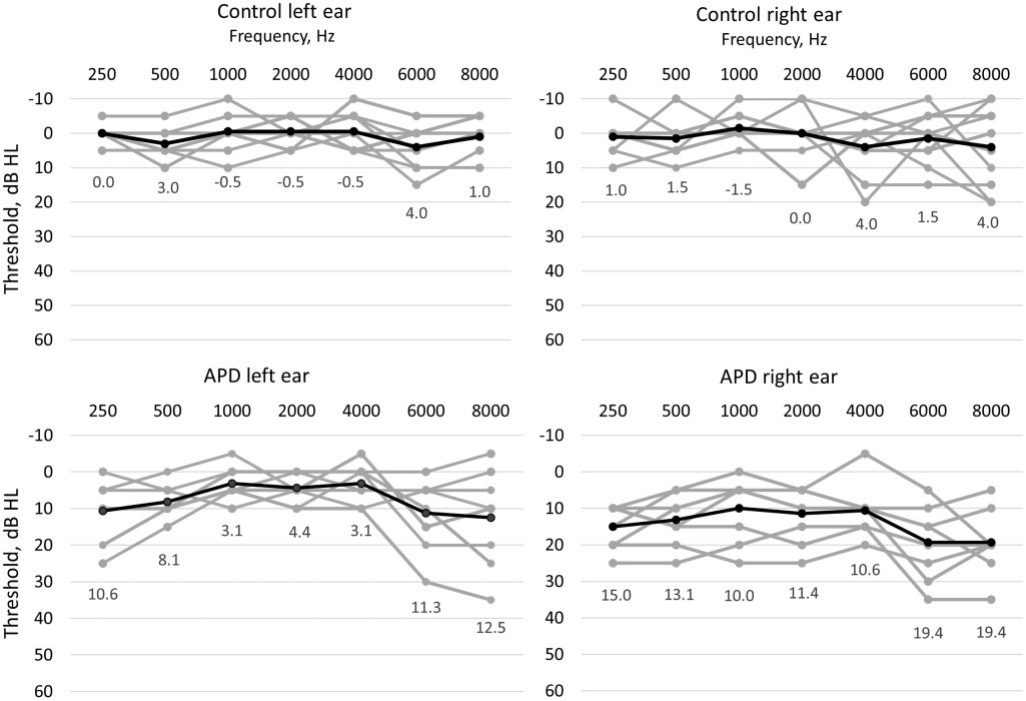
Individual (gray lines) and mean (black lines) pure-tone audiograms for the control group (top) and APD group (bottom) for the left ears (left) and right ears (right). The numbers within the panels indicate the mean for each frequency for that group/ear.

Figure 1 shows the individual and average audiograms for the two groups. All children in both groups had pure-tone thresholds within the normal range (20 dB HL or better) for audiometric frequencies from 250 to 8000 Hz in at least one ear. However, the APD group tended to have higher thresholds than the control group, especially at 6000 and 8000 Hz, and some of the APD group had thresholds worse than 20 dB HL in one ear for some frequencies. For the conventional pure-tone average threshold (PTA) across the audiometric frequencies 500, 1000, 2000, and 4000 Hz, an independent samples *t* test revealed the APD group had significantly poorer thresholds than the control group: *t*(16) = 5.58, *p* < .0001. For the APD group, the absolute value of the difference in PTA across ears ranged from 1.25 to 20 dB, with a mean of 8.0 dB. Three of the children (apd3, apd4, and apd8) had an interaural difference in PTA of 15 to 20 dB, while the interaural asymmetry was 7.5 dB or less for the remaining children. The three children with interaural asymmetry ≥15 dB performed relatively poorly on the dichotic words subtest (competing words [CW]) of the SCAN-3:C but did not show especially poor performance for the dichotic sentences subtest (CS).

### Signal Processing

**Figure 2.**
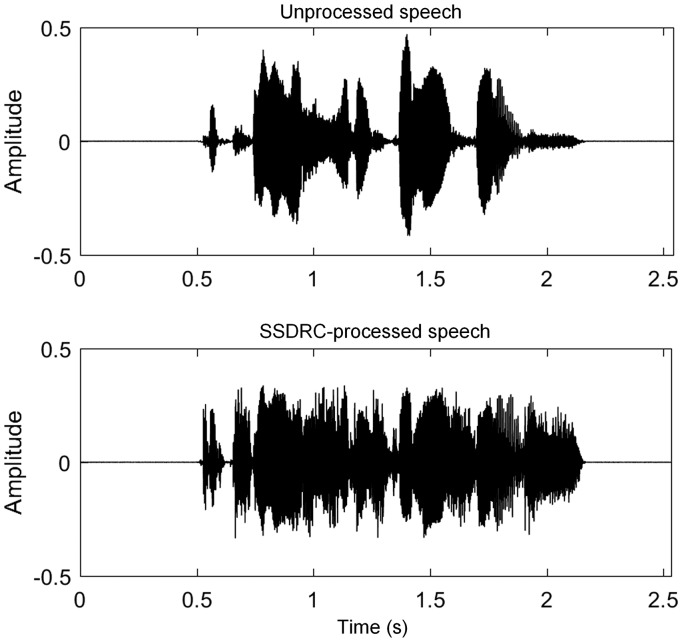
Example waveform of unprocessed speech and the same waveform after processing with SSDRC. The two sentences have the same root-mean-square level. The sentence was “The clown had a funny face.”.

The SSDRC algorithm had two processing stages, spectral shaping followed by time-varying amplitude compression. For full details, see Zorila et al. (2012). The spectral shaper was frame based and its operation was controlled by a measure of the strength of voicing in the current frame. The spectral shaper transferred energy from components with frequencies below 0.5 kHz to higher frequencies in such a way that the formants were sharpened, the spectral tilt was flattened, and the SBR in the range 0.5 to 4 kHz was increased. Dynamic range compression was applied to the broadband signal, with the aim of amplifying the weaker parts of speech that are more prone to masking (fricatives, nasals, and stops), while attenuating parts with more energy (vowels; Yoo et al., 2007). The loudness of the processed and unprocessed sentences was matched using the loudness model described by Glasberg and Moore (2002). This has been shown to be effective in equating the loudness of unprocessed and SSDRC-processed sentences (Zorila, Stylianou, et al., 2016). Figure 2 illustrates the effect of SSDRC processing for an example sentence “The clown had a funny face.”

### Procedure and Stimuli

During the experiment, the participant and the experimenter were seated within a sound-attenuating booth or audiometric room. The participant sat facing a computer screen, and opposite the experimenter. The participant listened to target sentences presented diotically over Sennheiser HD580 headphones, which have approximately a diffuse-field response. Sound levels are specified as equivalent diffuse-field levels.

The sentences were taken from the Bamford–Kowal–Bench sentences lists (Bench, & Bamford, 1979) and were spoken by a female native speaker of British English. The stimuli were played out from the audio output of a laptop computer equipped with a 16-bit soundcard and were amplified using an Aphex Headpod model 454 headphone amplifier. The system and amplifier output were calibrated at the start of each session so that the sentences were played at a comfortable level of 65 dB SPL. The participant was instructed to listen for the female voice and then repeat the sentence back to the experimenter. There were three or four keywords per sentence. The experimenter entered the correctly identified keywords directly into the computer using a silent touch-screen interface. The experimenter did not know whether or not a given sentence was processed using SSDRC. The participant received visual feedback on the monitor via reward points indicating the number of correct words reported and an unrelated jigsaw-like puzzle picture, which increased in pieces as the session progressed.

The sentences were mixed with one of two background sounds, either speech-shaped noise (SSN) or a competing speaker (CS), where the competing speaker was a male voice reading Harvard sentences. The background sound was turned on 0.5 s before the start of each target sentence and ended 0.5 s after the end of each sentence. Two SBRs were used for each background type, resulting in a total of eight conditions: unprocessed or SSDRC-processed speech, background SSN or CS, SBRs: 0 and −3 dB for SSN; −7 and −10 dB for CS (designated *High* and *Medium*). These SBRs were selected based on pilot experiments so as to give scores that were between about 30% and 90% correct, hence avoiding floor and ceiling effects.

The session started with eight practice sentences to familiarize the participant with the task and the SSN and CS backgrounds. The main experiment used 128 sentences. The experiment was designed to retain the attention of the participant. The experiment was divided into four blocks of 32 sentences, beginning with the easiest conditions and then increasing in difficulty in subsequent blocks. The SSN background was used in the first two blocks and the CS background was used in the other two. The SBR was high for Blocks 1 and 3, and medium for Blocks 2 and 4. The presentation order of processed and unprocessed stimuli within a block was balanced. For half of the participants, the first 16 sentences were processed and the other 16 were unprocessed. For the other half of the participants, the order was reversed. The overall session length was 1 hr, which included the audiometric testing and ample rest breaks.

## Results

**Figure 3.**
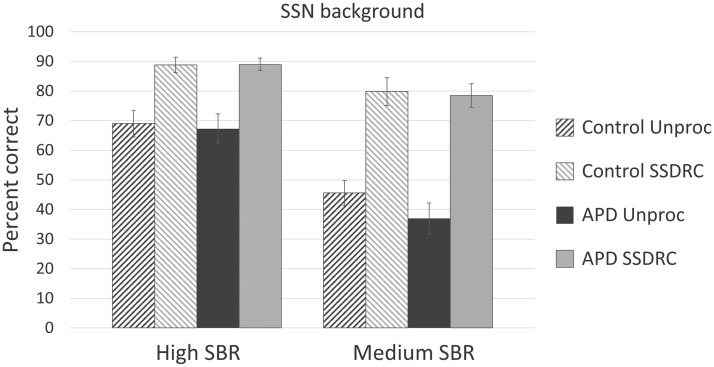
Average percentage correct keyword identification of sentences in SSN for the control participants (diagonal stripes) and APD participants (solid bars) without processing (Unproc, rising stripe/dark shading) and with processing (SSDRC, falling stripe/light shading), using high (left) and medium (right) SBRs. Error bars show ± 1 standard error.

**Figure 4.**
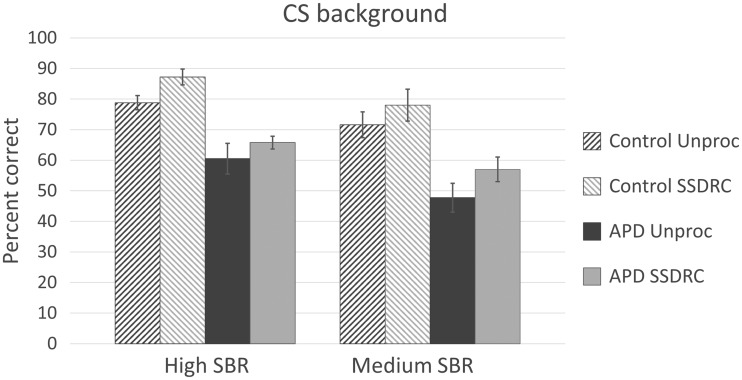
As Figure 3, but for the CS background.

The average scores are shown in Figure 3 (SSN background) and Figure 4 (CS background). Error bars show ± one standard error of the mean. As the SBRs differed for the two background types, a separate mixed-design analysis of variance (ANOVA) was performed on the arcsine-transformed proportion correct identification data for each background type, with within-subjects factors processing method (two values) and SBR (two values), and between-subject factor APD status (APD or control). Mauchley’s test showed that the condition of sphericity was satisfied for both ANOVAs.

For the SSN background, speech identification was significantly better with SSDRC processing than without: *F*(1, 16) = 179.05, *p* < .0001, ηp^2 ^= 0.842. As expected, there was a significant main effect of SBR: *F*(1, 16) = 39.50, *p* < .0001, ηp^2 ^= 0.712. There was no significant difference between the APD and the control group: *F*(1, 16) = 0.357, *p* = .559, ηp^2 ^= 0.022. There was significant interaction between SBR and processing method, *F*(1, 16) = 13.574, *p* < .01, ηp^2 ^= 0.459, reflecting the fact that the benefit of SSDRC processing was greater for the medium than for the high SBR. There were no other significant interactions.

For the CS background, speech identification was again significantly better with SSDRC processing than without, *F*(1, 16) = 6.116, *p* < .05, ηp^2 ^= 0.277, but the effect size was smaller than for the SSN background. As expected, there was a significant main effect of SBR: *F*(1, 16) = 9.594, *p* < .01, ηp^2 ^= 0.375. The control group performed significantly better than the APD group: *F*(1, 16) = 9.051, *p* < .01, ηp^2 ^= 0.361. There were no significant interactions.

On average, the control group showed similar performance for the SSN and for the CS backgrounds, despite the lower SBRs used for the latter. However, the APD group generally performed more poorly with the CS than with the SSN background, suggesting a specific problem with the CS. Also, the two groups had almost identical scores for the SSN background, but the APD group had significantly lower scores than the control group for the CS background.

The increase in percentage correct scores produced by SSDRC processing was calculated for each participant for each of the four combinations of background type (SSN, CS) and SBR (Mid, Hi). The mean increase with SSDRC processing was 29.1 percentage points (standard deviation, *SD* = 14.5) for the SSN background and 7.3 percentage points (*SD* = 16.0) for the CS background. A paired samples *t* test (two tailed) showed that the increase was significantly greater for the SSN than for the CS background: *t*(35) = 6.20, *p* < .0001.

As the APD group had significantly poorer audiometric thresholds than the control group, it was of interest to investigate if there was a relationship between each participant’s PTA and their word recognition scores. To assess this, two Pearson correlations were calculated, both based on scores for unprocessed speech and the medium SBR. The first correlation was between the PTA values and the scores with the SSN background and the second was between PTA values and scores with the CS background. The significance level was set to 0.025 to allow for the fact that two correlations were calculated. The first correlation was not significant (*r* = −0.36, *p* = .142). The second correlation was significant (*r* = −0.593, *p* = .01). This supports the idea that subtle peripheral dysfunction can affect the ability to understand speech in the presence of a complex background even when the audiogram remains within normal limits (Léger, Moore, & Lorenzi, 2012).

## Discussion

One limitation of this study was that one child (apd2) in the APD group had a low score only for the CW subtest of the SCAN3:C and three children (apd1, apd4, and apd6) had low scores only for two subtests of the SCAN3:C. The diagnosis of APD for these children must be considered as borderline. However, the scores for these four children on the speech tests conducted for the main experiment were well within the range of those for the other children, who had a firmer diagnosis of APD. A second limitation is that three of the children in the APD group (apd3, apd4, and apd8) had an interaural difference in PTA of 15 to 20 dB. However, the pattern of results for these three children was similar to that for the other children diagnosed with APD. Specifically, they showed a clear benefit of SSDRC processing for the SSN background but not for the CS background. Participants apd3 and apd4 showed slightly poorer performance than the average for the APD group, while Participant apd8 showed somewhat better performance than this average. Another limitation is that, on average, the APD group had poorer audiometric thresholds than the control group. This may have been true in previous studies, since details of the audiometric thresholds were often not reported. The possible consequences of the higher audiometric thresholds for the APD group are discussed further.

SSDRC processing resulted in a significant overall increase in intelligibility for both the APD and the control groups. The improvement for the APD group confirms the potential for the application of SSDRC processing in classroom situations. The signal picked up by a microphone close to the teacher’s mouth could be subject to SSDRC processing prior to transmission to wireless receivers worn by the child with APD. This should enhance the ability to understand the teacher’s voice when there was background noise in the classroom, as is often the case (Nelson & Soli, 2000).

The control and APD groups performed equally well with the SSN background. However, the APD group performed less well than the control group with the CS background. This suggests that the children with APD had a specific problem when the background was speech or when the background was fluctuating. Consistent with this, Middelweerd, Festen, and Plomp (1990) tested a group of adults with self-reported difficulties in understanding speech in the presence of background sounds but with normal or near-normal audiograms, and found that the difference in speech reception threshold (SRT) between this group and a control group without such difficulties was greater for a fluctuating background (SSN modulated by the envelope of a single talker) than for a steady SSN. Ferguson, Hall, Riley, and Moore (2011) compared SRTs for a group of children diagnosed with APD and a control group, using nonsense words and sentences presented in noise that was modulated with the envelope of a single talker. The mean SRTs were higher (worse) for the APD group than for the control group by about 4 dB for the nonsense words and 2 dB for the sentences. However, the differences between the two groups were not statistically significant. Lagacé, Jutras, Giguere, and Gagne (2011) found that children diagnosed with APD had poorer keyword recognition scores than a control group for sentences presented in a background babble. Overall, it seems likely that children with APD have specific problems in understanding speech in the presence of fluctuating background sounds, but further research is needed to establish more clearly the types of backgrounds that lead to difficulties in speech perception for children with APD.

Hearing is sufficiently developed by the start of the third trimester that the embryo can respond to sound (Kisilevsky, Pang, & Hains, 2000). However, some skills, such as amplitude-modulation detection (Hall & Grose, 1994) and frequency-modulation detection (Banai, Sabin, & Wright, 2011), continue to develop until the early teens. Hence, the problems displayed by children with APD might partly reflect delayed maturation (Tomlin & Rance, 2016). One of the biggest problems in measuring the auditory abilities of children, especially if they have a diagnosis of APD, is dissociating auditory perceptual problems from more general difficulties in performing the task. In this study, the APD group could clearly perform the task as well as the control group, as performance with the SSN background was almost identical for the two groups. The difference in performance across groups for the CS background could be explained by the following not mutually exclusive factors: (a) The APD group may have been less able to “listen in the dips” of the interfering speaker (Peters, Moore, & Baer, 1998), perhaps reflecting a problem in selecting “when to listen” (Hall, Buss, Grose, & Roush, 2012). (b) The APD group may have been more affected than the control group by “informational masking” from the CS (Brungart, Simpson, Ericson, & Scott, 2001), as has been found to be the case for children with dyslexia (Calcus, Colin, Deltenre, & Kolinsky, 2015). (c) The significantly higher audiometric thresholds of the APD group than of the control group might indicate a subtle deficit in cochlear functioning for the former, even though audiometric thresholds were within the normal range for at least one ear. The effects of cochlear dysfunction on speech identification have often been found to be greater for a CS background than for an SSN background (Baer & Moore, 1994; Duquesnoy, 1983; Peters et al., 1998) and, consistent with this, for the children tested here, the correlation of speech understanding scores with the PTA was significant for the CS background but not for the SSN background. Whatever the reason for the problems experienced by the APD group with the CS background, it seems clear that SSDRC processing has the potential to improve speech intelligibility in the presence of both noise and speech backgrounds, and therefore could be used to alleviate such problems in classroom situations.

## Appendix: SCAN-3:C Test for Auditory Processing Disorder

The SCAN-3:C test (Keith, 2000) is individually administered either in an audiometric booth or in a quiet room to children aged between 5 years and 12 years 11 months. The stimuli are played over headphones from a CD. The subtests are as follows:

- **Filtered words (FW):** The child is asked to repeat monosyllabic words that have been low-pass filtered at 750 Hz.
- **Auditory figure-ground (AFG).** The child is asked to repeat words that are presented against a background of multitalker speech babble at +8 dB SBR.
- **Competing words (CW).** Two monosyllabic words are presented simultaneously, one to each ear, and the child is required to repeat each word.
- **Competing sentences (CS).** Pairs of sentences are presented simultaneously, one to each ear. The child is asked to repeat the sentence presented to one ear while ignoring the other.
  **Table A1.** Scores for Each Participant in the APD Group on the SCAN3:C for Each Subtest and Overall.
  	FW	AFG	CW	CS	TCS	Overall
  apd1	10	9	6	6	8	7.8
  apd2	7	8	6	7	Not tested	7.0
  apd3	6	6	4	6	4	5.2
  apd4	8	5	5	8	Not tested	6.5
  apd5	7	8	5	4	1	5.0
  apd6	9	6	7	8	2	6.4
  apd7	5	2	4	4	1	3.2
  apd8	4	6	3	7	3	4.6
  *Note.* AFG = auditory figure-ground; CS = competing sentences; CW = competing words; FW = filtered words; TCS = time-compressed sentences.
- **Time-compressed sentences (TCS).** This is a supplementary test used to assess the ability to process degraded speech. Sentences are presented that have been time compressed by 60%. The child is instructed to repeat the sentences heard.

The total number of correct responses for each subtest and the mean across subtests are converted to standardized scores based on normative data from U.S. school children organized according to age band. The normal range is 7 to 13 points, with an average of 10. A score of 6 or below is considered to be below the normal range. Subtest and overall scores for each child in the APD group are given in Table A1.
